# Predictive Analysis of the Neutralization Activity in Convalescent Plasmas From COVID-19 Recovered Patients in Zhejiang Province, China, January-March, 2020

**DOI:** 10.3389/fcimb.2021.650487

**Published:** 2021-03-16

**Authors:** Yajie Yuan, Liang Yu, Zi Jin, Yongjun Wang, Meng Gao, Haojie Ding, Xunhui Zhuo, Xiao Zhu, Fei Gao, Xiaojun Zheng, Guoqing Ying, Xiaowei Xu, Qingming Kong, Shaohong Lu, Hangjun Lv

**Affiliations:** ^1^ Institute of Parasitic Diseases, Hangzhou Medical College, Hangzhou, China; ^2^ School of Biological Engineering, Hangzhou Medical College, Hangzhou, China; ^3^ State Key Laboratory for Diagnosis and Treatment of Infectious Diseases, National Clinical Research Center for Infectious Diseases, National Medical Center for Infectious Diseases, Collaborative Innovation Center for Diagnosis and Treatment of Infectious Diseases The First Affiliated Hospital, College of Medicine, Zhejiang University, Hangzhou, China; ^4^ Blood Center of Zhejiang Province, Hangzhou, China; ^5^ Guangdong Key Laboratory for Research and Development of Natural Drugs, The Marine Biomedical Research Institute, Guangdong Medical University, Zhanjiang, China; ^6^ Department of research and development, Hangzhou AllTest Biotech Co., Ltd, Hangzhou, China; ^7^ College of Pharmacy, Zhejiang University of Technology, Hangzhou, China

**Keywords:** SARS-CoV-2, convalescent plasma, neutralizing antibody, IgG antibody, indirect ELISA

## Abstract

**Background:**

Convalescent plasma (CP) transfusion is considered to be the priority therapeutic option for COVID-19 inpatients when no specific drugs are available for emerging infections. An alternative, simple, and sensitive method is urgently needed for clinical use to detect neutralization activity of the CP to avoid the use of inconvenient micro-neutralization assay.

**Method:**

This study aims to explore optimal index in predicting the COVID-19 CP neutralization activity (neutralizing antibody titers, NAb titers) in an indirect ELISA format. Fifty-seven COVID-19-recovered patients plasma samples were subjected to anti-SARS-CoV-2 RBD, S1, and N protein IgG antibody by indirect ELISA.

**Results:**

ELISA-RBD exhibited high specificity (96.2%) and ELISA-N had high sensitivity (100%); while ELISA-S1 had low sensitivity (86.0%) and specificity (73.1%). Furthermore, ELISA-RBD IgG titers and pseudovirus-based NAb titers correlated significantly, with R^2^ of 0.2564 (P < 0.0001).

**Conclusion:**

ELISA-RBD could be a substitute for the neutralization assay in resource-limited situations to screen potential plasma donors for further plasma infusion therapy.

## Introduction

Severe acute respiratory syndrome coronavirus 2 (SARS-CoV-2) is the causative agent of the novel emerging coronavirus disease 2019 (COVID-19), which has induced ongoing global threat after the first case reported in late December 2019 in Wuhan, China (Salata et al., 2019). The COVID-19 pandemic, due to the rapid human-to-human transmission by SARS-CoV-2 among almost all of counties and regions, has caused substantial mortality worldwide (Lai et al., 2020; Malik et al., 2020). To date, many countries have made great breakthrough in clinical drug trial, while the specific therapeutic treatments are still unavailable for clinical use for this evolving disease (Dehelean et al., 2020). Considering the situation of fast increasing infected patients, since the early of March, CP has been recommended for emergent use in treating severe COVID-19 patients in the most countries of the world (Chen and Xia, 2020; Mahase, 2020; Shen et al., 2020).

To evaluate the efficacy of CP, Li and his colleagues set a randomized controlled trial and found that CP (S-RBD specific IgG titer ≥ 1:640) was unable to shorten the time to clinical improvement of the severe and life-threatening COVID-19 patients (Li et al., 2020). It was reported that therapeutic effect of CP is associated with active agents, donor conditions, infusion time and other factors, most notably plasma quality (Franchini, 2020; Mehew et al., 2020). The higher the titer of NAb is, the better the CP quality will be. Nowadays, pre-donation screening for donors’ plasma with high NAb levels is recommended as an essential prerequisite before CP transfusion due to the observation of highly variable NAb titers in COVID-19-recovered patients (Ko et al., 2017; Robbiani et al., 2020; Wang et al., 2020).

NAbs are antibodies that can directly interfere with virus’ replication and prevent virus from entering target cells. In SARS-CoV-2 specific-NAbs, IgG and IgM are the predominant antibody followed by the IgA (Rojas et al., 2020; Schlesinger et al., 2020). Neutralizing antibody titers were in accordance with anti-SARS-CoV-2 IgG and IgM antibody titers (Figueiredo-Campos et al., 2020; To et al., 2020). Anti-SARS-CoV-2 IgM antibody reached to peak within 3 weeks and then began to decline, while IgG antibody remained elevated for a long time (Liu et al., 2020; Song et al., 2020). Therefore, in our study, we aimed to detect NAb titers by neutralization assay and anti-IgG titers by indirect ELISA assay.

Micro-neutralization assay is a “gold-standard” measuring assay for CP neutralization activity. CP quality can be identified either by directly testing NAb titers against live SARS-CoV-2 virus, or against pseudotype virus. But both two methods are highly labor-intensive and time-consuming, making them unsuitable for large-scale screening in clinical applications. In addition, the former method must be conducted in Biosafety level 3 (BSL-3) laboratories to prevent the contamination of live SARS-CoV-2 virus (Case et al., 2020; Lei et al., 2020). As for the latter method, there is no grid standard existing among different laboratories (Nie et al., 2020). Therefore, the availability of a simple and reliable serological assay to study and detect the immune response(s) to SARS-CoV-2 in a qualitative and quantitative manner is critical in CP therapy.

Recently, several scientists have discovered that ELISA, except for detecting SARS-CoV-2 infection, is capable of quantifying anti-SARS-CoV-2 antibody level (Shrock et al., 2020; Walls et al., 2020). However, little is known about the SARS-CoV-2-specific immune response and its relationship with NAb responses. A few reports have found that anti-SARS-CoV-2 NAb titers may exhibited some kind of relation with anti-RBD IgG antibody levels, but the correlation results between anti-N IgG antibody levels and NAb titers exhibited inconsistence (Ni et al., 2020; Okba et al., 2020; To et al., 2020). It remains uncertainty that whether ELISA reactivity is able to forecast neutralization activity of CP (Bloch et al., 2020).

SARS-CoV-2 genome encodes four main structural proteins: the spike protein (S), nucleocapsid protein (N), membrane protein (M) and envelop protein (E) (Flanagan et al., 2020). Among all of the SARS-CoV-2 proteins, the receptor-binding domain (RBD) located in the S1 subunit of S protein, which plays an important role in virus entry host cells *via* aiding in human angiotensin converting enzyme 2 (ACE2) receptor binding. N protein is a necessary protein for virus replication and proliferation (Amanat et al., 2020; Kaddoura et al., 2020).

Herein, we generated the RBD, S1 and N protein as coating antigens to detect IgG antibodies responses in COVID-19-recovered plasmas by using indirect ELISA. We found that RBD based ELISA (ELISA-RBD) possessed higher detection selectivity and N based ELISA (ELISA-N) showed higher sensitivity. The correlation analysis performed between SARS-CoV-2-specific IgG antibody levels and NAb titers was further proved that anti-RBD IgG antibody levels could serve as an index to predict NAb titers. To further improve the sensitivity of ELISA assay, we optimized the coating antigen by combining RBD and N protein with specific proportion, making the protocol suitable for high-throughput evaluation of plasma quality before CP transfusion.

## Materials and Methods

### Patients and Ethics

We conducted a cohort study focusing on the NAb titers and IgG antibody levels of CP samples. Serum samples were collected from 57 COVID-19-recovered patients in Zhejiang province from January 2020 to March 2020. Participated patients were selected based on the clinical treatment plan of COVID-19 convalescent plasma (trial second edition in China). Obtained Patients’ information was included age, sex, blood type, and clinical classification (classification according to the eighth edition of the guidelines on the diagnosis and treatment of COVID-19 by the National Health Commission, China).

The study was approved by the Ethics Commission of Clinical Research Ethics Committee of the First Affiliated Hospital, College of Medicine, Zhejiang University (2020-IIT-18).

### Indirect ELISA Test

Briefly, the recombinant antigens (RBD, S1 and N) were cloned into pET-28a vector, expressed as C-terminally His-tagged fusion in *Escherichia coli* system and purified by affinity chromatography. RBD、S1 and N proteins were diluted to 5 μg/mL in pH 9.6 10 mM carbonate buffer solution (CBS) and coated in 96-well ELSIA plate for 100 μL/well overnight at 4 °C to detect IgG antibodies. The antigen coated plates were then blocked with 5% non-fat milk (250 μL/well) in phosphate-buffered saline (PBS) containing 0.05% Tween 20 (PBST) for 2 h at 37 °C. Inactive treated plasma samples were diluted in blocking solution from 1:100 to 1:12800 (2-fold serially dilution), added to well (100 μL/well) and incubated for 1 h at 37 °C. Goat anti-human IgG-HRP were diluted to 1:5000 in blocking solution, added to plate (100 μL/well) and incubated for 30 min at 37 °C. Tetramethylbenzidine (TMB) substrate solution was added for 50 μL/well and then incubated 10-15 min at 37 °C. Finally, 2% H_2_SO_4_ (50 μL/well) was added to stop the reaction. The plates were washed with 10 mM PBST before each step. The optical density (OD) value was read by micro-plate reader (Bio-Tek company) at 450 nm (A450). The positive cut-off value was set as the mean A450 nm value for 26 negative samples plus 0.15.

For improving specificity and sensitivity of ELISA, RBD protein combined with N protein as co-antigen in different proportions at 1:1, 1:3, 1:5, 1:7, 3:1, 5:1, and 7:1. A random positive sample diluted to 1:200, 1:800, and 1:3200 was tested by ELISA performed as described above. OD value scatter diagram was drawn to analyze optimal proportion of the combination of RBD and N protein.

### Assay Repeatability Assessment

The uniformity and variability of the assay were validated by using intra-batch repeatability and inter-batch repeatability assay. The tests were performed by using random positive plasma diluted to 1:200. At least two independent experiments were conducted for inter-batch repeatability analysis. Each experiment used three ELISA plates to assess inter-plate variations. The intra-batch repeatability analysis was performed by using four replicates for each plasma sample group in one plate.

### Pseudovirus Neutralization Assay

Lentivirus-based SRAS-CoV-2 pseudovirus system was developed as described previously (Chu et al., 2018). In Brief, full-length Spike gene from strain Wuhan-Hu-1 (GenBank: MN908947.3) was codon-optimized for human cells and cloned into pcDNA3.1 to generate the recombinant plasmid pcDNA3.1-spike. The pcDNA3.1-spike and pNL4-3.Luc.RE plasmids were co-transfected 293FT cells using Lipofectamine 3000 transfection reagent. Supernatants were harvested and filtered at 48 h post-infection. The pseudovirus titers were then determined by measuring luciferase activity.

HEK293-ACE2 cells were seeded in 96-well cell culture plate at a density of 30,000 cells/well and cultured in 100 μL DMEM with 10% fetal bovine serum (FBS) at 37 °C under 5% CO_2_ atmosphere for 12 h. The 50 μL pseudovirus were pre-incubated with 5-fold serially diluted 50 μL heat-inactivated plasma at 37 °C for 1 h before adding to the cell. To detect viral infectivity, the plate was changed by 200 μL fresh culture medium after 12 h infection and continuously cultured for an additional 48-72 h. Cell lysates were then prepared and used directly to measure luciferase activity. In this experiment, each plate contained three experimental groups, including uninfected HEK293-ACE2 cells (negative control), pseudovirus-infected cells but untreated with CP (positive control), and pseudovirus-infected cells treated with different dilution of CP. At least two biological replicates were conducted for these groups. The cut-off value of pseudovirus neutralization assay was set as 30 of the IC_50_ value according to Nie et al. work, in which they established and validated a pseudovirus neutralization assay based on VSV pseudovirus system for SARS-CoV-2 (Nie et al., 2020). (This part was performed by Sino Biologicol in Beijing).

### Micro-Neutralization Assay

The Vero-E6 cells were seeded in 96-well cell culture plate at a 20,000 cells/well and cultured in 100 μL MEM with 10% FBS at 37°C under 5% CO_2_ atmosphere for 12 h. The samples were diluted at 1:10, 1:20, 1:40, 1:60, 1:80, 1:120, 1:160, 1:240, and 1:320. Healthy donor (HD) plasmas were used as negative control. SARS-CoV-2 viruses were diluted to 200 TCID_50_. Then 50 μL SARS-CoV-2 viruses mixed with 50 μL serially diluted 50 μL plasmas were pre-incubated at 37 °C for 1 h. The plasma-virus mix was added to the cells and co-cultured for 6 days at 35 °C. The titers were defined as the highest dilution of sample that demonstrated an inhibitory effect (>50%) on SARS-CoV-2. Three biological replicates were conducted for this assay (This part was performed by the First Affiliated Hospital, Collage of Medicine, Zhejiang University).

### Statistical Analysis

IBM SPSS 26.0 and Graphpad prism 8.4 software were exploited for statistical analysis and plotting. Measurement data were analyzed by mean and 95% confidence interval (CI). Enumeration data were analyzed by chi-square test. Comparisons between two groups were performed by unpaired t test. Linear regression was used for correlation analysis between IgG antibody levels and NAb titers. Value of P<0.05 was considered statistically significant.

## Results

### Variable Levels of SARS-CoV-2-Specific NAbs in CPs From COVID-19 Convalescent Patients

**Figure 1:**
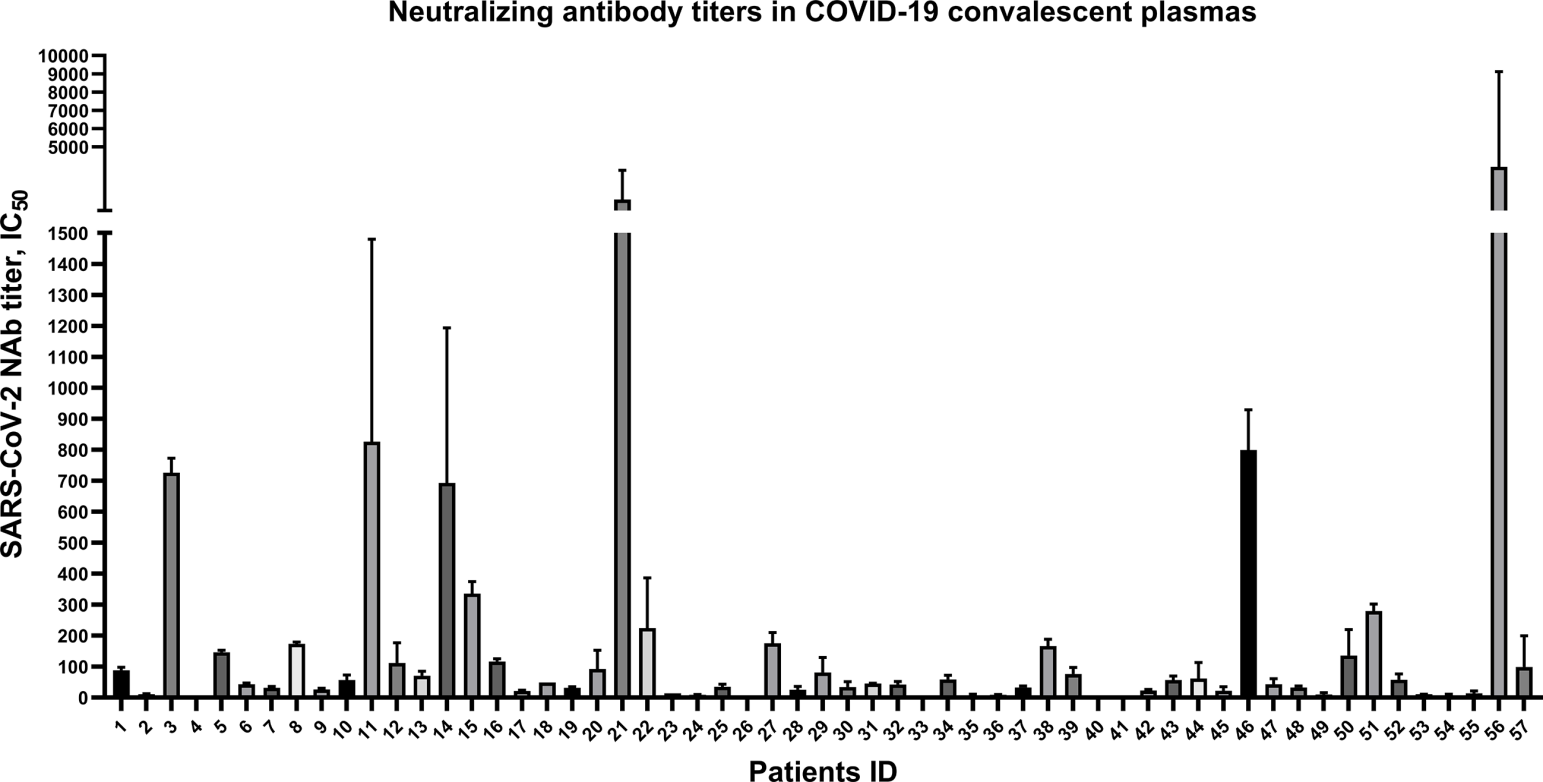
Highly variable neutralizing antibody titers of 57 convalescent plasmas from COVID-19 patients discharged in Zhejiang province from January to March in 2020. Plotted by IC_50_ mean with SD. Neutralizing antibody in 5 convalescent plasma samples were undetectable.

To explore the anti-SARS-CoV-2 NAb titers in 57 CP samples, spike-pseudovirus neutralization assay was conducted. In our cohort, SARS-CoV-2-specific NAb persisted in almost of CP samples, but the NAb titers were highly variable (i.e., ranging from 6 to 1093) (**Figure 1**). Among the 57 patients, there were only 3 severe cases and others were mild and ordinary cases (**Supplementary Material Table S1** and **Table S2**), which may be possible to draw this conclusion: the majority of mild and ordinary patients produced low level of NAb titers.

### Identification of IgG Antibody Against S1, RBD, N protein in CPs by Indirect ELISAs

**Figure 2:**
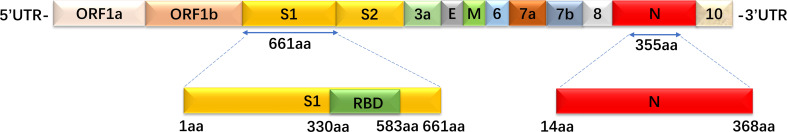
The recombinant S1, RBD, and N protein amino acid position mapping. These three proteins were used as coated target antigens to establish indirect ELISA to detect IgG antibody levels against SARS-CoV-2.

RBD, S1, and N proteins were generated as coating antigens for ELISAs (**Figure 2**). To confirm the sensitivity and specificity of IgG antibodies against RBD, S1 and N proteins, serially diluted CP samples and HD samples (from 1:100 to 1:12,800) were used to evaluate IgG responses against various viral proteins *via* OD450 values detection. Of the 57 CP samples, the sensitivities of the RBD, S1 and N proteins were 94.7% (54/57), 86% (49/57), and 100% (57/57), respectively. As of 26 HD samples, the specificities of the RBD, S1 and N proteins were 96.2% (25/26), 73.1% (19/26), and 88.5% (23/26), respectively.

**Figure 3:**
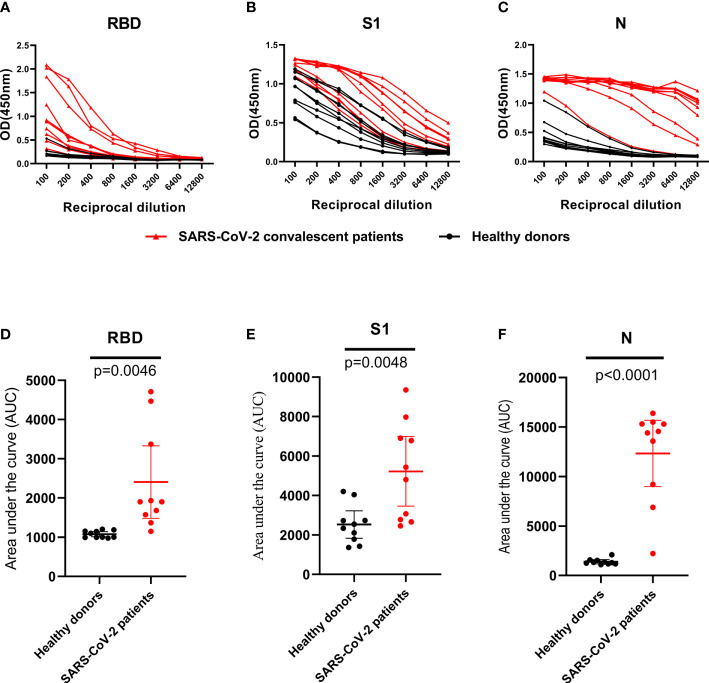
IgG antibodies response against RBD, S1 and N protein in control and SARS-CoV-2 convalescent plasma. **(A–C)**, OD 450 values of IgG antibodies against RBD, S1 and N protein in randomly selected healthy donors (n=10; black dot and line) and SARS-CoV-2 convalescent plasma (n=10; black triangle and red line). **(D–F)**, Data from the same samples in **(A–C)**, respectively, but plotted by AUC mean with 95% CI. Statistical analyses were performed using corresponding unpaired two-tailed Student’s t test. Horizontal lines represent mean values.

A set of 10 randomly selected samples from the CP were then used to establish dilution curves for determine the reactivity of plasma samples to viral proteins (**Figures 3A–C**). The area under the curve (AUC) analysis was performed by using the values from the curves (**Figures 3D–F**). Ten HD plasmas included as controls yielded background reactivity. Despite all of the RBD, S1 and N proteins were recognized by CP, there was stronger cross-reactivity observed between CP and HD groups in anti-S1 IgG response (**Figures 3B, E**), indicating that the S1 fragment of spike protein is not a specific antigen for SARS-CoV-2 diagnostics. However, we found, in our study, the background reactivity against RBD protein was lower than both N and S1 proteins, and included the previous finding of RBD protein exhibited high specificity in HD plasmas, suggesting that RBD protein is suitable to be used in SARS-CoV-2 diagnostics (**Figures 3A, D**). Additionally, our data showed that the reactivity of CP samples was stronger against N protein than that of S1 and the RBD proteins and revealed that ELISA coated with N protein exhibited higher sensitivity than other proteins (**Figure 3C**).

### Evaluation of Assay Repeatability

Two CP samples were selected randomly and detected with both intra-assay and inter-assay variation. The plasmas were diluted to 1:200 and four biological replicates were conducted for every sample. CV value was calculated for intra-assay variation and one-way ANOVA was used to evaluate inter-assay variation. The results suggested a minor intra-assay variation (CV < 2%) (**Table 1**) and inter-assay (F = 4.578, p = 0.123, p > 0.05) (**Table 2**). As a result, our indirect ELISA assay was reproducible.

**Table 1:** Intra- batch repeatability.

Reciprocal dilution	Repeat			Mean	Standard error	CV (%)
	1	2	3			
100	1.418	1.411	1.381	1.403	0.016	0.011
200	1.335	1.388	1.334	1.336	0.002	0.001
400	1.308	1.300	1.284	1.297	0.010	0.008
800	1.186	1.214	1.221	1.207	0.015	0.013
1600	1.066	1.068	1.046	1.060	0.010	0.009
3200	0.722	0.783	0.772	0.776	0.005	0.007

**Table 2:** Inter-batch repeatability.

Batch/OD value	Repeat		ANOVA
	1	2	
**First**	1.366	1.418	F=4.578
**Second**	1.311	1.284	P=0.123
**Third**	1.357	1.304	

### Comparison of the ELISA-IgG and Neutralizing Antibody

Since the RBD and N protein both showed higher specificity and sensitivity than S1 protein, we hypothesized that anti-RBD and anti-N IgG responses were able to be used as index to determine the neutralizing titers before CP transfusion. To confirm this hypothesis, we compared IgG antibody levels and NAb titers against viral proteins (RBD, S1 and N protein) by correlation analysis.

Fifty-seven CP samples were examined in parallel comparing a SARS-CoV-2 pseudovirus neutralization assay (PVN) and the indirect ELISAs mentioned above. In ELISA assay, 34 CP samples were found to be positive and 23 were negative. In the PVN test, 31 were positive and 26 were negative. In total, 28 CP samples were determined to be positive in both cases, and 7 were judged to be negative by both assays, which represented a consensus for 61.4% of samples. Three samples that were negative in the ELISA were positive in the PVN test, and nineteen samples that were positive in the ELISA were negative in the PVN test. The positive concordance rate (PC) of the ELISA in comparison with the PVN test was 56%, while the negative concordance rate (NC) was 24.1% (**Table 3**). In addition, the Kappa Cohen’s coefficient was calculated to evaluate the consistency between the ELISA and PVN test, and the result of 0.393 (p = 0.003, p < 0.05) indicates a good compatibility between two assays.

**Table 3:** Determination of the agreement of indirect ELISA for the detection of anti-SARS-CoV-2 IgG antibody to neutralization assay for detection of neutralizing antibody.

ELISA	PVN test		
	Positive	Negative	Total
**Positive**	28	19	47
**Negative**	3	7	10
**Total**	31	26	57

We set 30 of the IC_50_ value as the limit of detection of pseudovirus neutralization assay and 800 of RBD specific IgG titers as the limit of detection of ELISA test.

**Figure 4:**
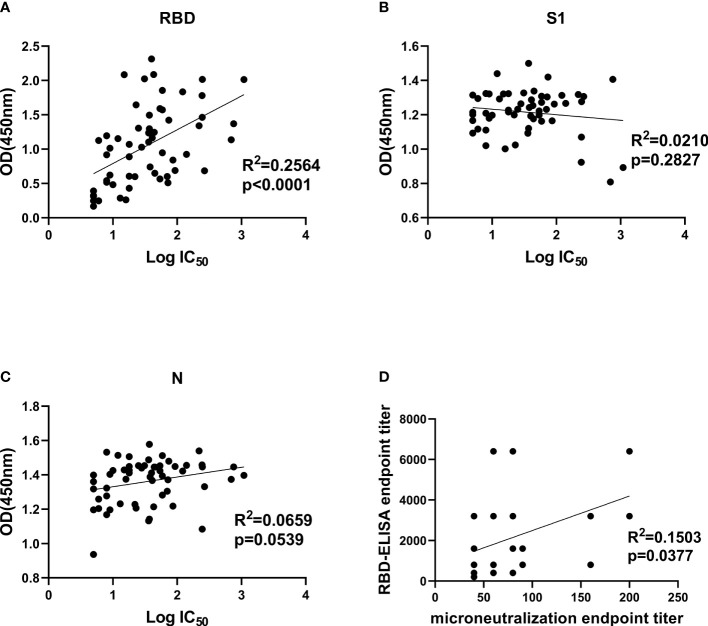
Correlation analysis between IgG antibody levels and neutralization antibody titers. **(A–C)**, Correlation analysis between neutralizing titers and RBD-, S1-, and N-specific IgG levels, respectively. Convalescent plasma collected from 57 COVID-19-recovered patients was used for SARS-CoV-2 pseudotype neutralization assay to test IC_50_ and indirect ELISA to test OD450 values at a fixed dilution (1:100). **(D)**, correlation analysis between authentic SARS-CoV-2 neutralizing antibody titers and anti-RBD IgG levels in convalescent plasma. Statistics analysis was performed by using Graphpad prism 8.4 software. IC_50_, half-maximum inhibitory concentration.

Comparison between NAb titers against SARS-CoV-2 pseudotype and anti-RBD, anti-S1 as well as anti-N IgG antibody levels was made to explore their correlation. It demonstrated that only anti-RBD IgG antibody levels exhibited a statistically significant correlation with pseudovirus NAb titers of patients’ plasmas (R^2^ = 0.2564, p < 0.0001), indicating that anti-RBD IgG antibody levels possibly could be used as monitoring index to determine neutralizing titers before CP transfusion (**Figure 4A**). Both anti-S1 and anti-N IgG antibodies were not related to pseudovirus NAb titers (**Figures 4B, C**). Moreover, we correlated the ELISA reactivity against RBD protein with neutralizing activity in COVID-19-recovered patients’ sera against SARS-CoV-2 Wuhan-Hu-1 strain. There is a moderate correlation (R^2^ = 0.1503, p = 0.0377) between anti-RBD IgG antibody levels and NAb titers against live virus (**Figure 4D**). The result of SARS-CoV-2 Wuhan-Hu-1 strain displayed consistency with of SARS-CoV-2 pseudotype, which proved that neutralizing activities were correlated with the IgG antibody response to SARS-CoV-2 RBD protein.

### Optimization the Sensitivity of RBD Protein

**Figure 5:**
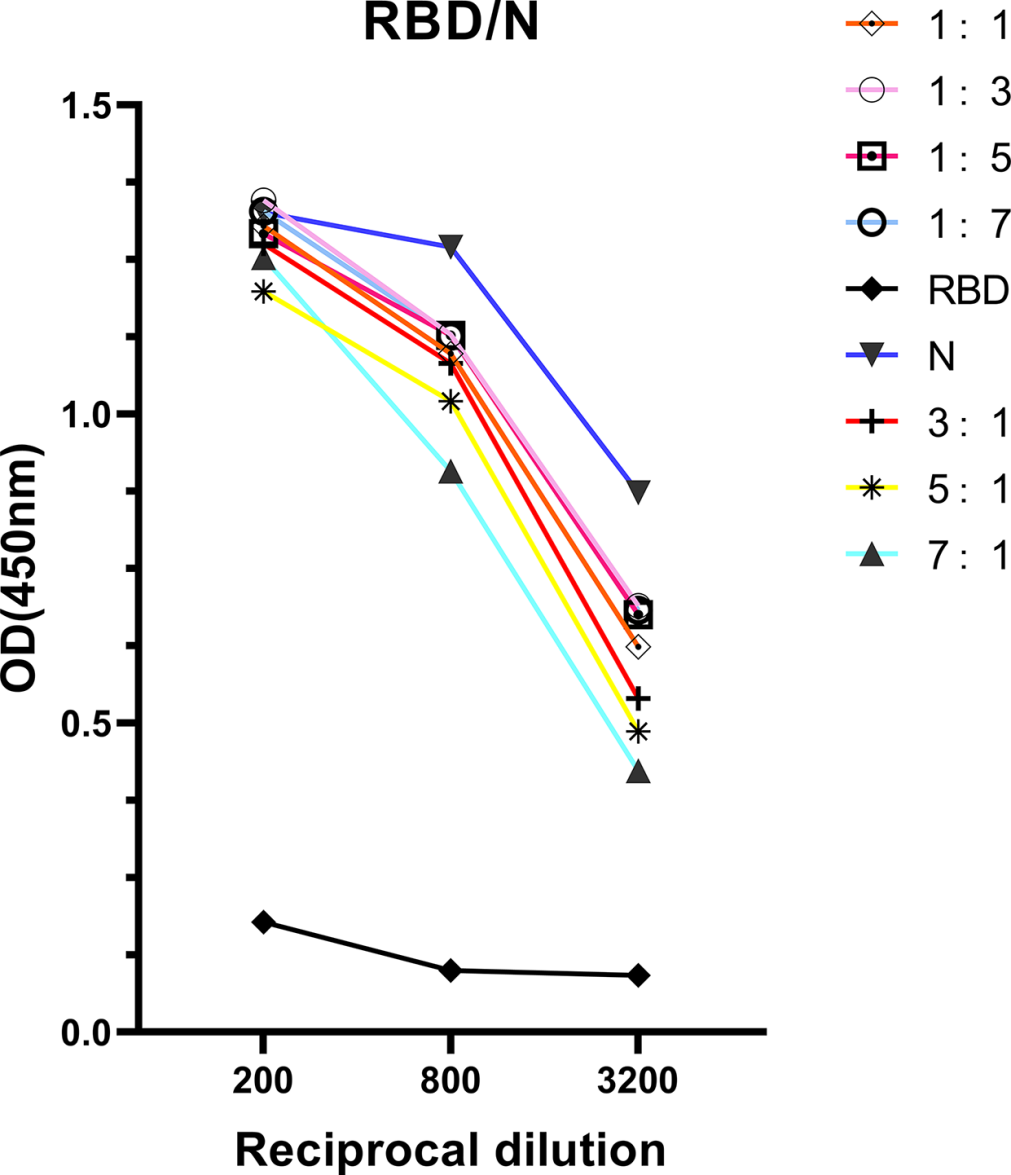
Antigenicity optimization for the combination of RBD protein and N protein. RBD protein was combined with N protein as co-antigen in different proportions at 1:1, 1:3, 1:5, 1:7, 3:1, 5:1, and 7:1, respectively.

The correlation found between anti-RBD IgG response and neutralizing activities indicated that detection of antibody response *via* ELISA could be useful for hospitals to screen optimal donors with high NAb titers in large- scale. However, the RBD protein has been proved obtaining lower sensitivity but higher specificity than N protein at the early of study. To improve the ELISA-RBD detection sensitivity without reducing its specificity, we hence performed experiments to determine the optimal ratio of joint combination of RBD and N proteins. The RBD-N combined protein was tested in different proportions (from 1:1, 1:3, 1:5, 1:7, 3:1, 5:1 to 7:1), RBD protein and N protein were used as controls. The RBD-N protein mixed at a specific ratio range (1:3 - 1:5) significantly improved the detection limit, compared with the RBD protein alone (**Figure 5**).

## Discussion

Convalescent plasma transfusion therapy has been successfully used to treat many infectious viral diseases. Neutralizing antibodies in the CP are important for virus clearance. Recently, a randomized controlled trial found that CP with S-RBD specific IgG titer ≥ 1:640 was unable to shorten the time to clinical improvement of the severe and life-threatening COVID-19 patients (Li et al., 2020). Similarly, another randomized controlled trial with an open label design from India reported that they didn’t measure the presence and NAb titers in the recipients before transfusion and found no effect of convalescent plasmas with a median titer of 1:40 on moderate COVID-19 mortality (Agarwal et al., 2020). Harkins’s work further proved these findings. They measured NAb and IgG titers in both donors and recipients before and after CP transfusion. This study founded that CP with low NAb titers had no impact on titers transfused recipients (Bradfute et al., 2020). It leads to the conclusion that screening NAb titers in both donors and recipients prior to infusion is important to choosing moderate donors according recipient status.

In this study, ELISA-RBD results showing high linear regression with NAb titers indicated that RBD is preferred to serve as index for forecasting NAb titers in CP. Considering the superior specificity for ELISA-RBD and sensitivity for ELISA-N, a better composition of RBD and N protein at a proportion from 1:3 to 1:5 was identified for the sensitivity improvement of ELISA-RBD.

S and N proteins are the two major immunogen antigens in SARS-CoV-2 virus (Luchsinger et al., 2020). RBD domain of the S1 subunit is essential for virus binding ACE2 receptor, while N protein participates in virus replication and proliferation (Dove et al., 2006; Yang et al., 2020). In our study, the stronger cross-reactivity on anti-S1 IgG response provided the evidence that S1 is not suitable for SRAS-CoV-2 diagnostics. Different finding was reported in previous study that S1 protein was preferred for SARS-CoV-2 diagnostics since no cross-creativity displayed with the S1 subunit (Okba et al., 2020). This is probably because some non-specific sites were involved in S1 protein when designed, which may explain the cause of strong cross-reactivity in our study. The study in cross-reactive antibody response also reported that sera from healthy donor samples reacted well with seasonal beta coronaviruses spike protein but not with RBD protein or S1 protein, while the CP from COVID-19 patients produced a signal against SARS-CoV-2 RBD protein and S1 protein (Amanat et al., 2020). As expected, RBD provided the best specificity, whereas N protein was more sensitive than RBD protein. It is possible because N protein is most conserved and informative among these three proteins, and its expression level is greater than S1 and RBD proteins during SARS-CoV-2 infection (Wang et al., 2005; Premkumar et al., 2020; Rongqing et al., 2020). This result, again, emphasized that N-based serological test is more sensitive than S protein, while RBD-based serological test is more specific (Haljasmagi et al., 2020).

Theoretically, only spike-specific antibodies can predict protective immunity in the patients, the correlation analysis showed the IgG level against RBD, but not against N and S1 proteins correlated with NAb titers. Our results in line with the report that antibody level against RBD protein was strongly related to NAb titers, instead of N protein (Ni et al., 2020). While others found IgG levels against N protein was strongly related to NAb titers (Okba et al., 2020; To et al., 2020). The inconsistent results about anti-N IgG antibody in different laboratories need further verification. Further, a striking correlation between anti-RBD IgG antibody and neutralizing activity was observed in COVID-19-recovered patients’ sera against SARS-CoV-2 Wuhan-Hu-1 strain. This is consistent with the theory that the RBD protein is the major region of protective antigen in S1 subunit of SARS-CoV-2 virus, which induced the direct production of the protection antibody (Lv et al., 2020).

Even though ELISA-RBD displayed strong correlation with neutralization antibody activity of the CP, it was proved obtaining lower sensitivity but higher specificity than N protein at the early of study. To improve the ELISA-RBD detection sensitivity without reducing its specificity, we briefly evaluated the possibility of using N protein combined with RBD protein as coating antigens for ELISA assay, and observed slight better performance than when using RBD protein alone. Our results indicated that RBD-N protein combined detection in neutralizing antibodies may be better than RBD protein detection, and this suppose needs further verification.

## Conclusion

Many countries have made great improvement in vaccine development; however, it is not widely available in resource-limited regions or countries. For now, there is no available COVID-19-specific drugs, CP is still the most promising therapeutic strategies for the treatment of SARS-CoV-2-infected patients. The application of neutralizing assay has been restricted in hospitals because of the several drawbacks, including complex operations, high cost, risk of contamination and others. Therefore, we proposed the application of ELISA-RBD as alternative assay and explored the relationship between immune reactivity and neutralizing activity of the CP. We found that the anti-RBD IgG antibody levels are positive correlated with NAb titers and it means that ELISA-RBD assay is capable to be used for predicting neutralization activity in the COVID-19-recovered patients and inpatients. ELISA is suitable for large-scale screening, can provide reliable quantification of antibody titers, which is beneficial for those resource-limited regions and hospitals to maximum therapeutic effect. Besides, it helps to understand immune status of the patients and thus provide reference for optimal treatment. However, due to the lack of strict standardization, it makes comparison between laboratories more difficult and explains why many poor clinical treatment effects. Overall, further validation and lab-to-lab variation are required.

## Data Availability Statement

The original contributions presented in the study are included in the article/**Supplementary Material**. Further inquiries can be directed to the corresponding authors.

## Ethics Statement

The studies involving human participants were reviewed and approved by Clinical Research Ethics Committee of the First Affiliated Hospital, College of Medicine, Zhejiang University. The patients/participants provided their written informed consent to participate in this study.

## Author Contributions

YY wrote the paper and analyzed the data. QK and XZ conceived of and designed the study. ZJ reviewed and edited the paper. YY, LY, XX, HD, XHZ, MG, FG, and XJZ performed the experiments. SL and HL coordinated the samples. YW provided the samples. QK, GY and HL administrated the project and funding. All authors contributed to the article and approved the submitted version.

## Funding

This work was sponsored by the Key R&D projects of Zhejiang province (2019C03057 to HL), the Collaborative Innovation Center of Yangtze River Delta Region Green Pharmaceuticals and the National Engineering Research Center for Process Development of Active Pharmaceutical Ingredients (QK and GY) and the Zhejiang Provincial Program for the Cultivation of High-level Innovative Health Talents (WJW2021002 to QK).

## Conflict of Interest

Authors FG and XJZ were employed by the company Hangzhou AllTest Biotech.

The remaining authors declare that the research was conducted in the absence of any commercial or financial relationships that could be constructed as potential conflict of interest.
