# Supplementary material for: Predictive Analysis of the Neutralization Activity in Convalescent Plasmas From COVID-19 Recovered Patients in Zhejiang Province, China, January-March, 2020
Source: Front Cell Infect Microbiol. 2021 Mar 16;11:650487. doi: 10.3389/fcimb.2021.650487 (PMC8008148; doi:10.3389/fcimb.2021.650487)
Supplement: Supplementary file 1 [file DataSheet_1.docx]

| Clinical information | | Pseudovirus NAb titers | | |
| --- | --- | --- | --- | --- |
|  | | <30 (N=26) | >30 (N=31) |  |
| Male（%） | | 12 (46.2%) | 13 (41.9%) |  |
| Female (%) | | 14 (53.8%) | 16 (53.6%) |  |
| Blood type Number (%) | A | 4 (15.4%)  3 (11.5%)  5 (19.2%)  14 (53.9%) | 9 (29.0%)  8 (25.8%)  4 (12.9%)  8 (25.8%) |  |
|  | B |  |  |  |
|  | AB |  |  |  |
|  | O |  |  |  |
| Median age [IQR] | | 28 [32.5-40.5] | 38.5 [33-43] |  |
| Median NAb titers [IQR] | | 16.5 [6.5-18] | 562 [42-179.5] |  |
| Anti-RBD IgG levels [IQR] | | 500 [250-1400] | 3200 [800-3200] |  |
| Mild disease (%) | | 6 (23.1%) | 5 (16.1%) |  |
| Ordinary disease (%) | | 17 (65.4%) | 17 (54.8%) |  |
| Severe disease (%) | | 0（0%） | 3 (9.7%) |  |

**Table S1**. Clinical statistics

**Abbreviations:** IQR, interquartile rang; Pseudovirus NAb titers, SARS-CoV-2 pseudotype neutralizing antibody titers;

**Table S2.** Clinical characteristics of donors

| **Donors' ID** | **Gender** | **Age** | **Blood type** | **Clinical classification** |
| --- | --- | --- | --- | --- |
| 1 | Female | 35 | AB | Mild |
| 2 | Female | Not tested | AB | Not tested |
| 3 | Not tested | Not tested | Not tested | Not tested |
| 4 | Male | Not tested | O | Not tested |
| 5 | Female | 39 | O | Ordinary |
| 6 | Female | Not tested | A | Not tested |
| 7 | Female | 30 | AB | Mild |
| 8 | Female | 24 | A | Mild |
| 9 | Male | Not tested | AB | Not tested |
| 10 | Male | Not tested | A | Not tested |
| 11 | Male | 31 | O | Ordinary |
| 12 | Male | Not tested | A | Not tested |
| 13 | Male | Not tested | O | Not tested |
| 14 | Female | 35 | A | Ordinary |
| 15 | Male | 44 | A | Ordinary |
| 16 | Male | 34 | O | Ordinary |
| 17 | Male | 32 | O | Ordinary |
| 18 | Female | 36 | AB | Ordinary |
| 19 | Male | 35 | O | Ordinary |
| 20 | Female | 29 | B | Mild |
| 21 | Female | 38 | B | Ordinary |
| 22 | Female | 49 | B | Ordinary |
| 23 | Female | 26 | O | Mild |
| 24 | Female | 37 | B | Ordinary |
| 25 | Male | 39 | O | Ordinary |
| 26 | Male | 34 | O | Ordinary |
| 27 | Male | 43 | O | Ordinary |
| 28 | Male | 49 | AB | Ordinary |
| 29 | Male | 50 | A | Severe |
| 30 | Male | 33 | AB | Mild |
| 31 | Female | 37 | B | Ordinary |
| 32 | Female | 33 | O | Mild |
| 33 | Female | 54 | A | Ordinary |
| 34 | Male | 32 | B | Severe |
| 35 | Female | 40 | O | Ordinary |
| 36 | Female | 37 | B | Ordinary |
| 37 | Female | 36 | O | Ordinary |
| 38 | Female | 49 | A | Ordinary |
| 39 | Male | 31 | A | Mild |
| 40 | Female | 43 | A | Ordinary |
| 41 | Female | 23 | A | Mild |
| 42 | Female | 39 | O | Ordinary |
| 43 | Not tested | Not tested | Not tested | Not tested |
| 44 | Female | 33 | AB | Ordinary |
| 45 | Male | 43 | O | Ordinary |
| 46 | Female | 51 | O | Ordinary |
| 47 | Female | 54 | B | Severe |
| 48 | Male | 44 | A | Ordinary |
| 49 | Female | 41 | B | Ordinary |
| 50 | Male | 29 | AB | Mild |
| 51 | Female | 39 | O | Ordinary |
| 52 | Female | 33 | B | Ordinary |
| 53 | Male | 34 | O | Ordinary |
| 54 | Female | 40 | O | Ordinary |
| 55 | Male | 21 | O | Mild |
| 56 | Male | 42 | B | Ordinary |
| 57 | Male | 29 | O | Ordinary |
